# Herbert Godfrey Eycott

**Published:** 1929

**Authors:** 


### Obituary.

### HERBERT GODFREY EYCOTT,

*Assistant Librarian in the Medical Library of the University of Bristol.*

THE death of Mr. Herbert Godfrey Eycott has deprived the University of the services of an Assistant Librarian whose work in the Medical Library was highly appreciated by the members of the Medico-Chirurgical Society. Mr. Eycott was appointed to the Medical Library in October, 1924, after holding a post in the Library of the Royal College of Physicians, London. He came, therefore, equipped with a wide knowledge of medical literature which he willingly placed at the disposal of readers in our library. The part he played in the production of this journal might pass unrecognized if the editorial committee were not to acknowledge his valuable help in proof reading, sending out and collecting books for review, and more especially in verifying references in original articles. The writers of most of the articles in this journal owe to Mr. Eycott an accuracy in quotation and reference which may sometimes have surprised themselves no less than it impressed their readers.

Mr. Eycott, who died at the early age of forty years, was educated in Leeds, and shortly afterwards took a position in connection with the railways of the south of Spain. At the beginning of the War he joined the Yorkshire Regiment and was twice wounded, on the second occasion so severely as to lead to the amputation of his right leg.

He bore his disability patiently and gallantly, and his final illness gave further evidence of his wonderful fortitude in the face of grievous pain and suffering. He died on June 5th, deeply mourned by all who knew him, leaving a widow to whom we offer our deepest sympathy.

